# Supplementary material for: The effects of secretome of umbilical cord mesenchymal stem cells on regeneration of sciatic nerve defects in Sprague dawley rats
Source: PLoS One. 2024 Dec 31;19(12):e0310467. doi: 10.1371/journal.pone.0310467 (PMC11687769; doi:10.1371/journal.pone.0310467)
Supplement: Attachment — Submitted filename: 20240723 - Response to Reviewer.docx [file pone.0310467.s001.docx]

Manuscript PONE-D-24-15970

Title: The Effects of Secretome of Umbilical Cord Mesenchymal Stem Cells on Regeneration of Sciatic Nerve Defects in Sprague Dawley Rat

Dear Editor and Reviewers,

We are pleased to be given a chance to revise our manuscript and to be considered in your journal. We have revised the manuscript according to your inputs in the response to reviewers’ table below.

Should you have any further inquiries, please kindly let us know.

Thank you for your kind attention.

Sincerely,

Ade Martinus

Corresponding Author

| Reviewers’ comments | Author’s responses | Changes made in manuscript |
| --- | --- | --- |
| **Reviewer #3** | | |
| 1. Line 93, the author should evaluate the content of CM. | Unfortunately we also isolated the CM, but did no evaluate the content. | - |
| 2. Line 109-111, the author should add the references of SFI evaluation. | Suggestion has been added to the manuscript. (reference 3, 9 and 10) |  |
| 3. Line 122, the authors should add the explanation how they prepare the histological sections. | Suggestion has been added to the manuscript. (line 132-135) | *The initial step on dehydration process was done using three grades of alcohol solutions of 70%, 90% and 100%, followed by immersion in benzyl alcohol and benzol-histoplast before moulded in paraffin block. The block was cut axially in 3µm then fixed onto the object glass before stained.* |
| 4. Line 128-129, the author should add the explanation to measure the axon number and diameter, and also add the references of the methodology. | Suggestion has been added to the manuscript. (line 138-141) | *..and examined sequentially with 40x magnification and then the fields underwent photomerge into 1 whole image through the PTGui Pro 9.1 program which was then carried out calculation analysis and recording by computer using the image-J program. The diameter of myelinated axons with toluidine blue staining was calculated by randomly taking 10 myelinated axons from each isolated ischiadic nerve sample.* |
| 5. Line 144, the author should add Table 1. A few values are on the manuscript. I cannot judge the statistical results. | The table has been added as an additional item to the submission | Table 1 |
| 6. Line 157, the order of the pictures in Figure 2 is different from the group oreder. The author should arrange the picture order according to group number. | Suggestion has been made and the picture has been in order of the group allocation. | Fig.2 |
| 7. Line 165-166, the value of the ration is rough. The author should recalculate the ratio down to third decimal. |  |  |
| 8. Line 177, the pictures in Figure 5 are rough. The author should add the picture in high magnification. They should also add the electron microscopical picture. | We don’t perform any electron microscopic on the specimen. |  |
| 9. In Materials and Methods, the author should add the experiment how secretom works. SFI, muscle weight and morphological study are not enough to explain UC-CM stimulates peripheral nerve regeneration. | Suggestion has been added to the manuscript. (line 94-100) | In a clinical context, the translation of umbilical cord secretome for peripheral nerve regeneration holds significant potential. Studies have highlighted the therapeutic applications of secretome in promoting nerve regeneration in animal models, indicating a promising avenue for clinical manifestation. The use of secretome from umbilical cord-derived mesenchymal stem cells has shown efficacy in supporting nerve regeneration and reinnervation in animal models, suggesting its potential for clinical use in peripheral nerve injuries. Furthermore, the immunomodulatory effects and safety profile of MSC-derived secretome have been investigated, providing valuable insights into the feasibility of utilizing secretome-based therapies in clinical settings (Nugrahaningsih et al., 2023). |
| 10. Figure 1, there is no explanation for the fourth picture. | Suggestion has been added to the figure legend | …. end result of silicon conduit placement) |
| 11. Figure 2, the author should arrange the picture order according to group number. | Suggestion has been made and the picture has been in order of the group allocation. |  |
| 12. Figure 4, the author should arrange the picture order according to group number. The regenerated nerve cannot be recognized in B, C or D. | Suggestion has been made and the picture has been in order of the group allocation. |  |
| 13. Figure 5, the pictures are rough. It is impossible to count the axons in those pictures. The author should add the picture in high magnification. They should also add the electron microscopical picture. | We didn’t carry out any observation using electron microscopical hence the absence of such image. |  |
|  |  |  |
| **Reviewer #2** | | |
| 1. The discussion could be enriched using the following reference:  The Long-term Effects of Uncultured Omental Adipose-derived Nucleated Cells Fraction and Bone-marrow Stromal Cells on Sciatic Nerve Regeneration  Saeed Azizi; Behnam Heshmatian; Saman Mahmoudpour; Abbas Raisi | Suggestion has been added to line 246-254 | Similar study on the effect on the effect of stem cells on nerve regeneration was carried out by Azizi. The study compared the long-term effects of uncultured omental adipose-derived nucleated cells (OADNCs) and undifferentiated bone marrow stromal cells (BMSCs) on sciatic nerve regeneration in rats. Both cell types were loaded into inside-out vein grafts bridging a 10-mm sciatic nerve defect. Functional, electrophysiological, morphometric, and immunohistochemical assessments showed no significant differences between the OADNC and BMSC groups in nerve recovery, electromyography amplitude, or time delay, despite both being superior to the control group. The lack of significant difference is attributed to the similar capabilities of OADNCs and BMSCs in enhancing nerve regeneration, suggesting that both cell types are equally effective for this purpose. |
